# Magnetic, Structural and Spectroscopic Properties of Iron(II)-Octacyanoniobate(IV) Crystalline Film Obtained by Ion-Exchange Synthesis

**DOI:** 10.3390/ma13133029

**Published:** 2020-07-07

**Authors:** Wojciech Sas, Dawid Pinkowicz, Marcin Perzanowski, Magdalena Fitta

**Affiliations:** 1 Institute of Nuclear Physics Polish Academy of Sciences, Radzikowskiego 152, 31-342 Krakow, Poland; Wojciech.Sas@ifj.edu.pl (W.S.); Marcin.Perzanowski@ifj.edu.pl (M.P.); 2 Faculty of Chemistry, Jagiellonian University, Gronostajowa 2, 30-387 Kraków, Poland; dawid.pinkowicz@uj.edu.pl

**Keywords:** molecular magnets, thin films, octacyanometallates

## Abstract

Over recent years, investigations of coordination polymer thin films have been initiated due to their unique properties, which are expected to be strongly enhanced in the thin film form. In this work, a crystalline [Fe^II^(H_2_O)_2_]_2_[Nb^IV^(CN)_8_]∙4H_2_O (1) film on a transparent Nafion membrane was obtained, for the first time, via ion-exchange synthesis. The proper film formation and its composition was confirmed with the use of energy dispersive X-ray spectroscopy and infrared spectroscopy, as well as in situ Ultraviolet-Visible (UV-Vis) spectroscopy. The obtained film were also characterized by scanning electron microscopy, X-ray diffraction, and magnetic measurements. The [Fe^II^(H_2_O)_2_]_2_[Nb^IV^(CN)_8_]∙4H_2_O film shows a sharp phase transition to a long-range magnetically ordered state at *T*_c_ = 40 K. The 1 film is a soft ferromagnet with the coercive field *H*_c_ = 1.2 kOe. Compared to the bulk counterpart, a decrease in critical temperature and a significant increase in the coercive field were observed in the films indicating a distinct size effect. The decrease in *T*_c_ could also have been related to the possible partial oxidation of Fe^II^ ions to Fe^III^, which could be efficient, due to the large surface of the thin film sample.

## 1. Introduction

Over the past few years, there has been significant interest in the study of molecular magnets in the form of thin films. Low-dimensional assemblies have assumed remarkable importance due to their unique properties, which make them attractive for future applications, for example, as chemical or photomagnetic sensors or in spintronic devices [1,2]. Among the group of cyanido–bridged coordination networks, the compounds that are studied the most are thin films of hexacyanidometallates, Prussian blue analogues (PBAs) [3,4], which can be fabricated using the electrodeposition [5,6,7] Langmuir–Blodgett technique [8,9], and multi-sequential adsorption [10,11]. Thin films of cyanometallate magnets can also be achieved using the ion-exchange method, where transition metal ions coordinate with hexacyanometallate anions leading to the formation of microcrystals on the surface of ion-sieving membrane. One of the examples of films of this type is hexacyanochromate-based magnetic film, M^II^_1.5_[Cr^III^(CN)_6_]·zH_2_O (M = Co, Ni, Cu), deposited on a Nafion membrane showing a Faraday effect in the visible region [12].

Among all reports, details on the preparation of thin films based on octacyanidometallates are somewhat limited and have mostly been related to Langmuir–Blodgett films [13]. Compared to PBAs, the polynuclear cyanido-bridged systems based on octacyanides of Nb, Mo, W, and 3d metal complexes constitute a relatively new and largely unexplored family of molecular assemblies. Coordination networks based on octacyanides are a highly desired class of coordination polymers for magnetic film preparation in terms of their unique properties such as porosity [14,15], photomagnetism [16,17], and magnetocaloric effect [18]. One of the strategies for constructing polynuclear systems is the “self-assembly” approach. It relies on the “building blocks”, i.e., cationic complexes of 3d and 4f metals and the anionic cyanidometallates. The molecular structure of the resulting polynuclear assembly is controlled by the number of labile coordination sites at the cationic complex available for substitution with the N-donor of the cyanido ligand. The stereochemical non-rigidity of the octacyanidometallate ion [M(CN)_8_]^n−^ (M = W^V^, Mo^V^, Nb^IV^) permits the construction of the coordination skeletons of different dimensionality such as one-dimensional (1D) chains, two-dimensional (2D) layers, and three-dimensional (3D) networks [19,20,21,22]. In addition, the careful selection of cationic building blocks permits the formation of discrete molecules, which is hardly observed in the case of hexacyanidometallate building blocks.

In this paper, we present the synthesis and characterization of crystalline [Fe^II^(H_2_O)_2_]_2_[Nb^IV^(CN)_8_]∙4H_2_O (1) film. The bulk counterpart of the (1) film with *T*_c_ ≈ 43 K was, for the first time, obtained and characterized by D. Pinkowicz et al. [23]. The (1) film is a soft ferromagnet. Ferromagnetic coupling between Fe^II^ (*S*_Fe_ = 2) and Nb^IV^ (*S*_Nb_ = 1/2) centers mediated by cyanido bridges results in the magnetization of saturation equal to 9 μ_B_/mol at *T* = 2 K. The [Fe^II^(H_2_O)_2_]_2_[Nb^IV^(CN)_8_]∙4H_2_O film, which is the subject of this paper, is the first example of an octacyanometallate-based molecular film prepared using the ion-exchange method.

## 2. Materials and Methods

### 2.1. Film Synthesis

All reagents used in this study were purchased from commercial sources (Sigma-Aldrich) and used without further purification. Potassium octacyanoniobate (IV) dihydrate K_4_[Nb(CN)_8_]∙2H_2_O was prepared according to the literature procedure [24]. The [Fe^II^(H_2_O)_2_]_2_[Nb^IV^(CN)_8_]∙4H_2_O film was prepared by ion-exchange synthesis. In the first step of the film preparation, a Nafion^®^ 117 membrane (1 × 2 cm) was immersed in an aqueous solution of FeCl_2_ (100 mM) for 5 min. Then, it was rinsed with deionized (DI) water and placed in an aqueous solution of K_4_[Nb(CN)_8_]∙2H_2_O (20 mM), independently, for 10, 30, 60, and 180 s. The deposition of [Fe^II^(H_2_O)_2_]_2_[Nb^IV^(CN)_8_]∙4H_2_O film started immediately, as evidenced by the change in membrane color. In the final step, the film was rinsed with deionized (DI) water and dried in ambient condition.

### 2.2. Characterization Techniques

The X-ray diffraction (XRD) patterns were obtained using a Panalytical X’PERT PRO diffractometer (Malvern Panalytical, Almelo, Netherlands) equipped with Cu anode and operated at 40 kV and 30 mA. The dried membranes were mounted on a silicon zero background diffraction plate. All the spectra were recorded in Bragg–Brentano geometry, at room temperature and normal pressure.

The infrared (IR) spectra were recorded on an EXCALIBUR FTS 3000 Fourier Transform Infrared (FTIR) spectrometer (BIO RAD, Hercules, CA, USA) using the transmission mode in the region 4000–400 cm^−1^.

The UV-Vis absorption spectra were recorded for the Nafion membrane containing Fe^II^ ions positioned in a quartz cuvette filled with an aqueous solution of K_4_[Nb(CN)_8_]·2H_2_O using an UV-VIS-NIR DH-2000 Micropack light source (Ocean Optics, Largo, FL, USA) and an USB2000 detector (Ocean Optics, Largo, FL, USA). UV-Vis spectra were acquired in situ every 1 s.

Microstructure and composition analysis of films was performed using a Tescan Vega 3 scanning electron microscope (TESCAN, Brno–Kohoutovice, Czech Republic ) equipped with an X-ray energy dispersive spectrometer EDAX Bruker (BRUKER, Karlsruhe, Germany). EDAX analysis (Fe/Nb ratio): calcd. 2; found: 2.03.

Magnetic properties were measured using a Quantum Design MPMS-XL magnetometer (Quantum Design, San Diego, CA, USA). For magnetic measurements, the samples deposited on Nafion^®^ 117 (FuelCellsEtc, College Station, TX, USA) were cut into pieces ca. 3 × 4 mm in size, and subsequently, they were introduced into the sample holder oriented parallel to the applied magnetic field. For reference, the magnetic properties of the Nafion^®^ 117 substrate were measured independently under the same conditions, and then subtracted from the raw data. Alternating current (AC) susceptibility was measured with a frequency of 1, 10, 100, and 1000 Hz and the oscillating field amplitude of 3.5 Oe.

## 3. Results and Discussion

**Figure 1:**
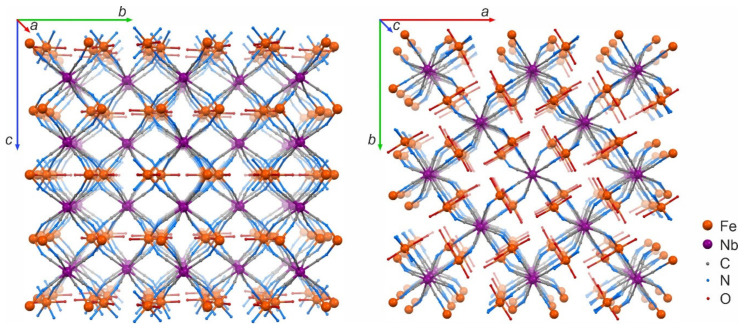
Packing diagrams presenting the crystal structure of {[Fe^II^(H_2_O)_2_]_2_[Nb^IV^(CN)_8_]·4H_2_O}_n_ as viewed along the *a* (**left**) and *c* (**right**) crystallographic direction (crystallization water molecules and hydrogen atoms omitted for the sake of clarity).

{[Fe^II^(H_2_O)_2_]_2_[Nb^IV^(CN)_8_]·4H_2_O}_n_ crystallizes in the tetragonal system with space group *I*4/m [23]. In this compound, octacyanoniobate anions are connected via cyanide bridges with the Fe cations, forming a 3D coordination framework, as depicted in Figure 1. Each Nb center is connected to four Fe and each Fe is connected to four Nb centers.

**Figure 2:**
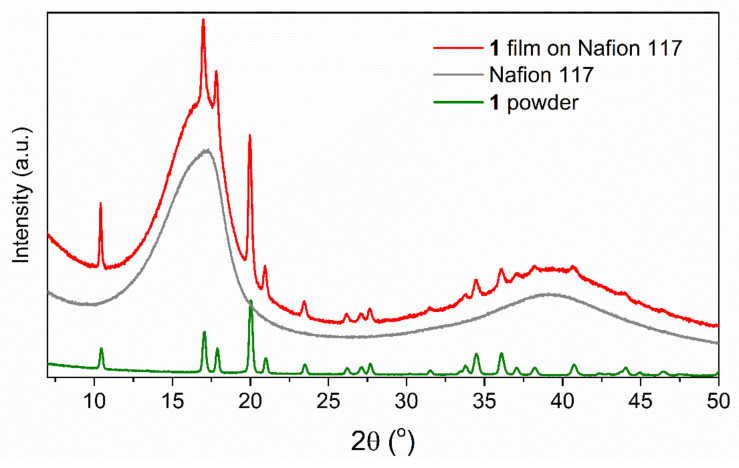
The XRD pattern measured for the Nafion^®^ 117 membrane, {[Fe^II^(H_2_O)_2_]_2_[Nb^IV^(CN)_8_]·4H_2_O}_n_ film deposited on the Nafion^®^ 117 and {[Fe^II^(H_2_O)_2_]_2_[Nb^IV^(CN)_8_]·4H_2_O}_n_ bulk sample.

Figure 2 shows the X-ray diffraction (XRD) data for the Nafion^®^ 117 membrane, {[Fe^II^(H_2_O)_2_]_2_[Nb^IV^(CN)_8_]·4H_2_O}_n_ film deposited on the Nafion^®^ 117, and {[Fe^II^(H_2_O)_2_]_2_[Nb^IV^(CN)_8_]·4H_2_O}_n_ powder sample. The XRD pattern for the Nafion^®^ 117 shows two characteristic broad signals at angle 2*θ*, i.e., 17° and 40°, which are related to the perfluorocarbon chains of the ionomer [25,26]. The diffraction pattern of the {[Fe^II^(H_2_O)_2_]_2_[Nb^IV^(CN)_8_]·4H_2_O}_n_ film deposited on the Nafion membrane reveals additional peaks, and the positions are fully consistent with the XRD pattern of {[Fe^II^(H_2_O)_2_]_2_[Nb^IV^(CN)_8_]·4H_2_O}_n_ powder. Therefore, we conclude that the crystal structure of the (1) film obtained in the form of the film and the powder is identical. Additionally, the film did not reveal preferred crystallite orientation.

**Figure 3:**
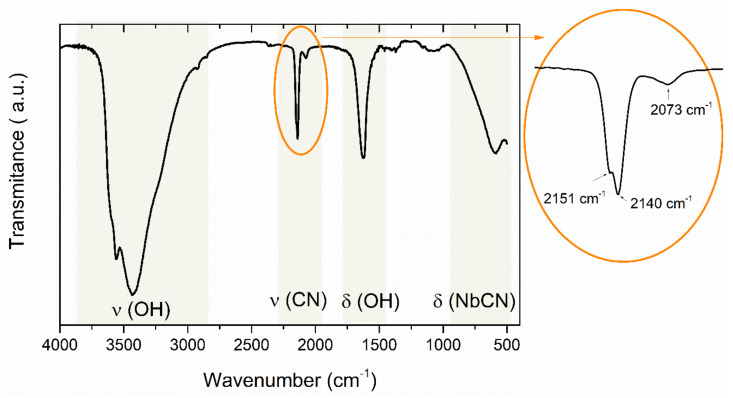
FT-IR spectra of [Fe^II^(H_2_O)_2_]_2_[Nb^IV^(CN)_8_]·4H_2_O recorded in KBr pellets with a tentative band assignment.

The formation of the cyanometallate network was confirmed by the FTIR spectroscopy and the measured IR spectra agree with earlier results reported for a bulk compound. The IR spectrum of the (1) film (Figure 3) exhibited two bands assigned to stretching (3576 cm^−1^) and bending (1635 cm^−1^) vibrations of O–H. The formation of the cyanidoniobate network is clearly supported by the ν (C≡N) stretching vibrations of the cyanide groups which are observed in the range 2090–2120 cm^–1^. Compared to the bulk sample, the spectra obtained for the film display one additional peak located at 2073 cm^−1^ which suggests possible slight oxidation of the sample.

**Figure 4:**
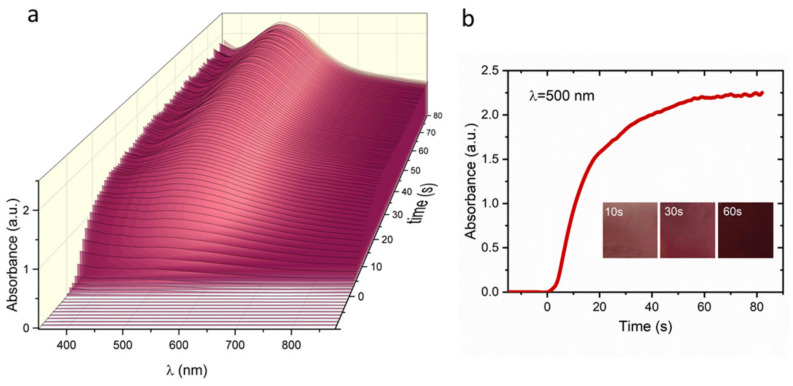
(**a**) UV-visible spectra obtained for [Fe^II^(H_2_O)_2_]_2_[Nb^IV^(CN)_8_]·4H_2_O film during the ion-exchange synthesis; (**b**) Time dependence of the absorbance of the film at the wavelength of 500 nm.

The process of the film’s growth was monitored in situ using UV-Vis spectroscopy. In the first step of this experiment, a Nafion^®^ 117 membrane (1 × 3 cm^2^) was immersed in an aqueous solution of Fe^II^Cl_2_ for 5 min. Then, it was rinsed with DI water and transferred to the cuvette containing an aqueous solution of K_4_[Nb(CN)_8_] 2H_2_O and the recording of UV-visible spectra was started. After a few seconds of the reaction, the homogeneous coverage of both sides of the Nafion membrane was observed. The color change from colorless to light violet within 10 s of ion-exchange synthesis indicated the start of film formation. An intense band observed around 500 nm was assigned to the following metal-to-metal charge transfer (MMCT) transition: Fe^II^–NC–Nb^IV^ → Fe^III^–NC–Nb^III^ (Figure 4a). The intensity of this band initially increases exponentially with time, while 30 s after that it tends to attain a constant absorption value as the transmitted light approaches the detection limit of the spectrometer due to the increasing film thickness. (Figure 4b).

**Figure 5:**
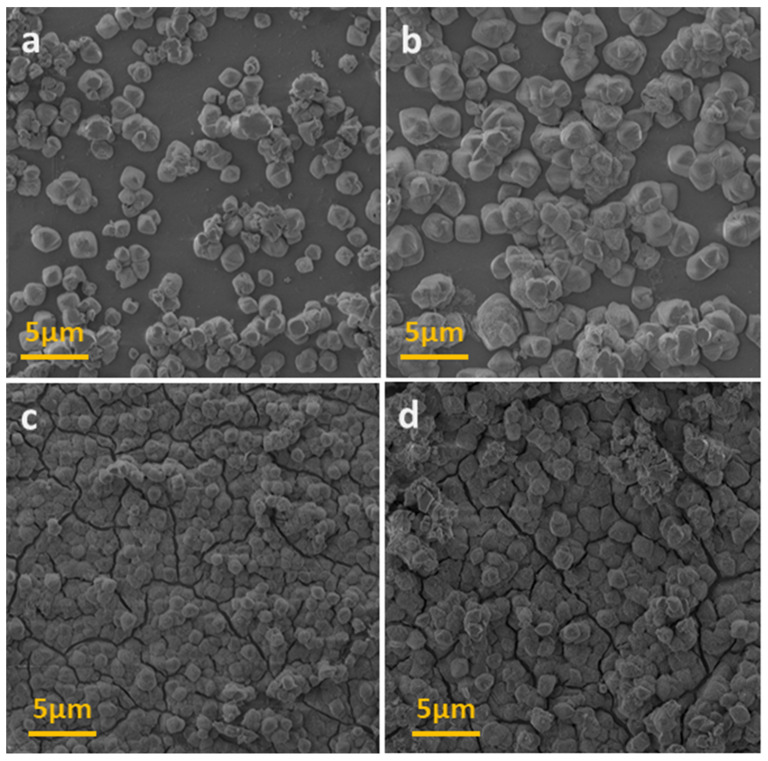
The SEM images of [Fe^II^(H_2_O)_2_]_2_[Nb^IV^(CN)_8_]·4H_2_O film obtained after 10 s (**a**); 30 s (**b**); 60 s (**c**); and 180 s (**d**).

In order to analyze the surface morphology, the films obtained with different times of ion-exchange reaction were characterized by SEM. Figure 5 shows the growth process of the {[Fe^II^(H_2_O)_2_]_2_[Nb^IV^(CN)_8_]·4H_2_O}_n_ film. After 10 s, the surface of the Nafion membrane was covered with octahedron-type crystals with a size of ca. 1 µm (Figure 5a). After 30 s, the crystals are much bigger (3 µm) and highly agglomerated. For samples obtained with the deposition time longer than 60 s, the total coverage of the Nafion surface is observed; the particles lack proper shape and are distributed densely on the surface. The cross-sectional view of the thin films shows rough surface morphology (Supplementary Materials Figure S3). The thickness of the films obtained with the synthesis times of 60 and 100 s are equal to ca. 5 µm and ca. 7 µm, respectively.

**Figure 6:**
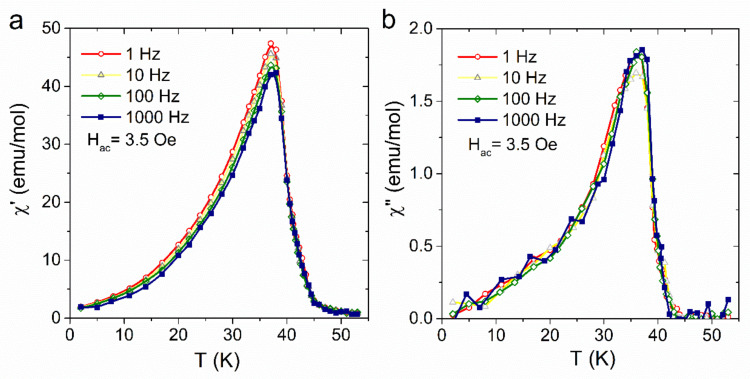
Temperature dependence of real (**a**) and imaginary (**b**) component of AC susceptibility of [Fe^II^(H_2_O)_2_]_2_[Nb^IV^(CN)_8_]·4H_2_O film.

Magnetic properties were measured for the samples with a thickness of 5 µm. The temperature dependence of the real and imaginary components of the AC susceptibility recorded for the film is presented in Figure 6. A significant *χ*’, *χ*” increase is observed upon cooling below 40 K, which indicates the transition to the long-range ordered state. The values of the critical *T*_c_ temperature determined as the minima on the d *χ*’/d*T* curves are equal to 40 K. This *T*_c_ value is lower than the critical temperature measured for related 3D bulk samples (*T*_c_ = 43 K) [23].

The AC susceptibility measured for {[Fe^II^(H_2_O)_2_]_2_[Nb^IV^(CN)_8_]·4H_2_O}_n_ film exhibits weak frequency dependence. The relative variation of the peak temperature per frequency decade $\Delta T_{p}/\left[T_{p}\Delta log\left(f\right)\right]$ is 0.005 which is in the range expected for canonical spin glasses.

**Figure 7:**
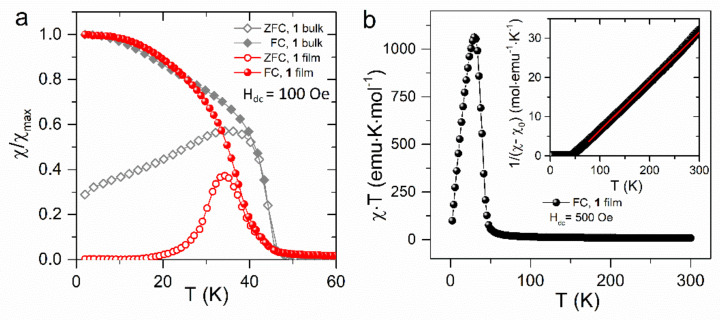
(**a**) Thermal variation of the DC susceptibility measured for (1) film and (1) bulk under zero field cooled (ZFC) and field cooled (FC) conditions in the applied field of 100 Oe; (**b**) Temperature dependence of *χ*·*T* for (1) film. Inset, thermal variation of the reciprocal magnetic susceptibility.

Figure 7a shows the temperature dependence of Direct current (DC) magnetization of the film samples measured in the field cooled (FC) and zero field cooled (ZFC) regime. The results obtained for the bulk samples are included for comparison. The bifurcation temperature of the ZFC and FC branches measured at 100 Oe is slightly less than *T*_c_. The irreversibility in the temperature dependence of the magnetization was also observed for the bulk samples, yet the differences between the ZFC and FC branches were smaller. Taking into account the frequency dependence of the AC susceptibility, the differences between the ZFC and FC data could be related to a spin-glass-like character. However, as discussed in [27], the essential reason for the irreversible behavior of ZFC and FC magnetization is the altered domain-wall motion under the different cooling conditions. The other factor that should be taken into account is the structural disorder, probably more pronounced in the case of the thin film as compared with the typical bulk sample. With the reduction of the grain’s size, disorder and frustration at the grain’s surface become progressively dominant with a tendency to form a spin glass phase and possible partial oxidation of Fe^II^ ions to Fe^III^, which could be efficient due to the large surface of the thin film sample.

The temperature dependence of the *χT* product is shown in Figure 7b. The high temperature *χT* limit of 7.8 emu K/mol is close to 7.635 emu K/mol expected for two high-spin ions Fe^II^ (*S* = 2), assuming *g*_Fe_ = 2.2 and one Nb^IV^ (*S* = 1/2) ion assuming *g*_Nb_ = 2. Fitting the Curie–Weiss law to the experimental data in the form of *χ*^−1^(*T*) gives Weiss temperature *θ* = 48.1 K and Curie constant *C* = 7.99 emu K/mol.

**Figure 8:**
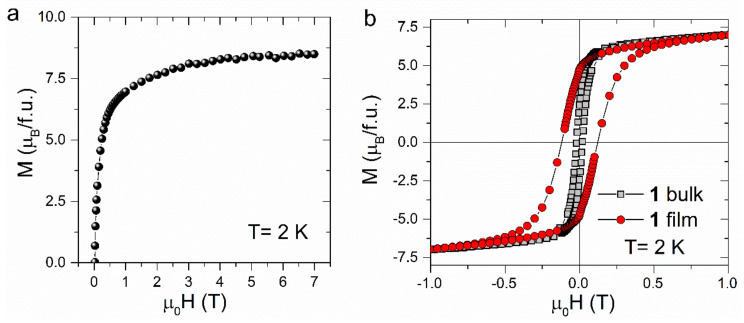
M(H) dependence (**a**) and hysteresis loop (**b**) measured for (1) film at *T* = 2 K. The hysteresis plot obtained for the bulk sample is included for comparison.

The results of magnetization vs. field measurements are shown in Figure 8. The field dependence of the magnetization measured at 2 K (Figure 8a) shows a fast increase to reach a value of ca. 7 µ_B_ in an applied field of 1 T, followed by a smooth increase to 9 µ_B_ at 7 T. As in the case of polycrystalline sample, the value of the magnetization at saturation is lower than 9.8 µ_B_ expected for two high-spin Fe^II^ (*g* = 2.2) and one Nb^IV^ (*g* = 2.0). This situation can be explained by the structural disorder, as well as the anisotropic character of the ferrous ions.

The low-field region of the hysteresis loops measured for {[Fe^II^(H_2_O)_2_]_2_[Nb^IV^(CN)_8_]·4H_2_O}_n_ film and its bulk counterparts are shown in Figure 7b. The coercive field determined for the film is equal to 1.2 kOe, which is a value that is one order of magnitude higher than that observed for bulk. The increase of coercivity in the film originates from the particle size effect. A similar observation has been previously described in other molecule-based materials, for example, electrodeposited thin films of Prussian blue analogues [6].

## 4. Conclusions

This contribution presents a successful and viable method for the preparation of molecular assemblies based on octacyanidoniobate and transition metal complex in the form of a thin film. The film was obtained with the use of ion-exchange synthesis on the transparent Nafion membrane. The proper film formation and its composition were confirmed with the use of EDS and IR techniques, as well as in situ UV-Vis spectroscopy. Our results show that the film’s structural and spectroscopic properties are compatible with its bulk counterpart. Significant differences between the film and the powder sample can be seen in the magnetic properties. Magnetic measurements reveal the critical temperature lowering and increase in the coercive field upon material downsizing. The [Fe^II^(H_2_O)_2_]_2_[Nb^IV^(CN)_8_]∙4H_2_O film is the first example of an octacyanometallate-based molecular film. The presented research opens up a new perspective for the preparation of thin film molecular assemblies.

## Supplementary Materials

The following are available online at https://www.mdpi.com/1996-1944/13/13/3029/s1, Figure S1: Schematic illustration of the growth process of [Fe^II^(H_2_O)_2_]_2_[Nb^IV^(CN)_8_]·4H_2_O crystalline film during ion-exchange synthesis, where a transparent Nafion membrane is used as a solid substrate., Figure S2: Energy dispersive X-ray spectroscopy (EDS) analysis data of [Fe^II^(H_2_O)_2_]_2_[Nb^IV^(CN)_8_]·4H_2_O film deposited on the Nafion membrane during ion- exchange synthesis with the time 60 s., Figure S3: The cross-section of the [Fe^II^(H_2_O)_2_]_2_[Nb^IV^(CN)_8_]·4H_2_O film deposited on the Nafion membrane during ion-exchange synthesis with the time (a) 10 s, (b) 30 s, (c) 60 s, and (d) 180 s.
