# Supplementary material for: Magnetic, Structural and Spectroscopic Properties of Iron(II)-Octacyanoniobate(IV) Crystalline Film Obtained by Ion-Exchange Synthesis
Source: Materials (Basel). 2020 Jul 7;13(13):3029. doi: 10.3390/ma13133029 (PMC7372376; doi:10.3390/ma13133029)
Supplement: Supplementary file 1 [file materials-13-03029-s001.pdf]

Supporting Information:

Magnetic, Structural and Spectroscopic Properties of Iron(II)-Octacyanoniobate(IV) Crystalline Film Obtained by Ion-Exchange Synthesis

Wojciech Sas [1], Dawid Pinkowicz [2], Marcin Perzanowski [1] and Magdalena Fitta [1,*]

1 Institute of Nuclear Physics Polish Academy of Sciences, Radzikowskiego 152, 31-342 Krakow, Poland; Wojciech.Sas@ifj.edu.pl (W.S.); Marcin.Perzanowski@ifj.edu.pl (M.P.)
2 Faculty of Chemistry, Jagiellonian University, Gronostajowa 2, 30-387 Kraków, Poland; dawid.pinkowicz@uj.edu.pl
* Correspondence: Magdalena.Fitta@ifj.edu.pl

Received: 27 May 2020; Accepted: 2 July 2020; Published: date

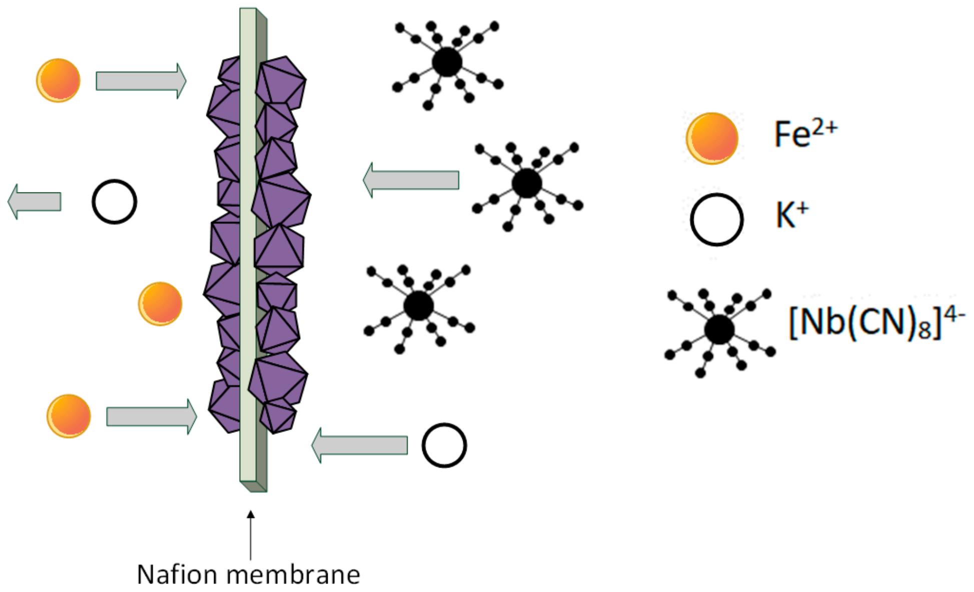

Figure S1. Schematic illustration of the growth process of $[Fe^{II}(H_2O)_2]_2[Nb^{IV}(CN)_8]\cdot 4H_2O$ crystalline film during ion exchange synthesis, where a transparent Nafion membrane is used as a solid substrate.

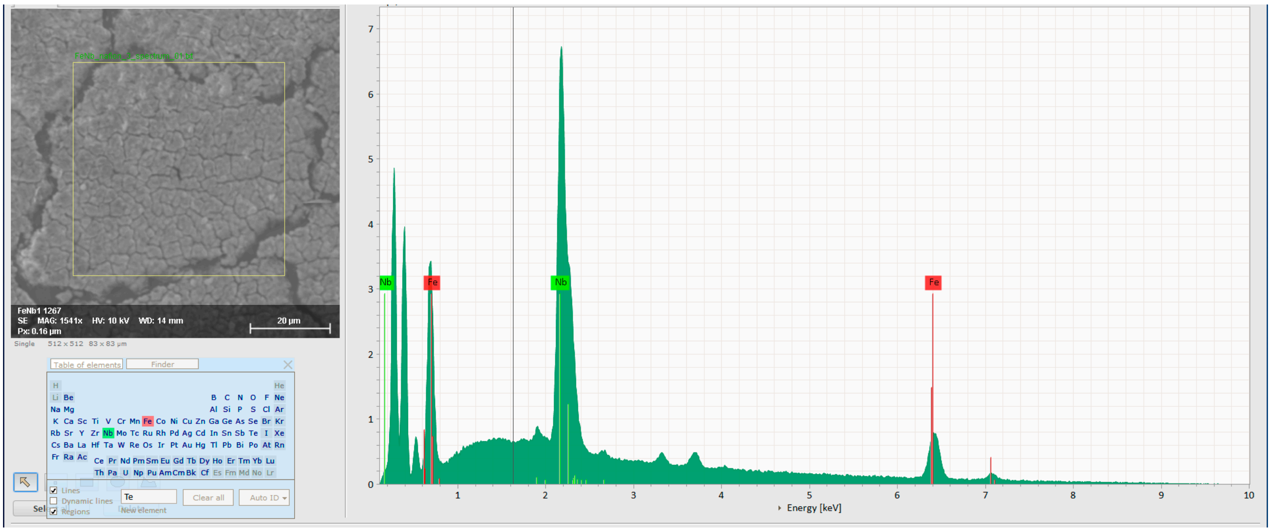

Figure S2. Energy dispersive x-ray spectroscopy (EDS) analysis data of $[Fe^{II}(H_2O)_2]_2[Nb^{IV}(CN)_8]\cdot4H_2O$ film deposited on the Nafion membrane during ion- exchange synthesis with the time 60s.

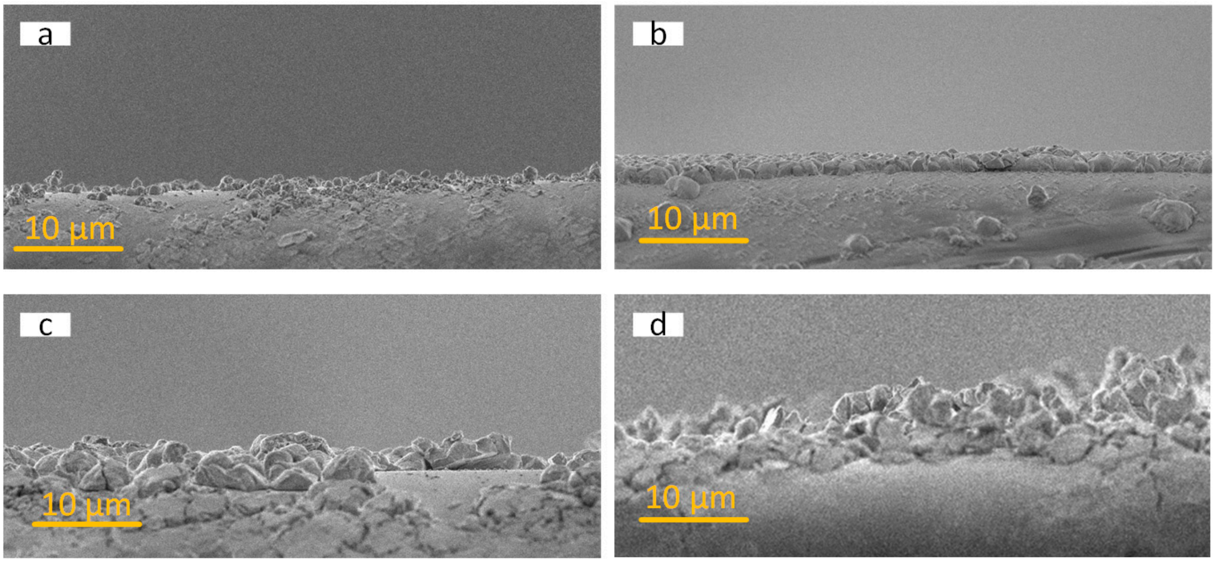

Figure S3. The cross section of the $[Fe^{II}(H_2O)_2]_2[Nb^{IV}(CN)_8]\cdot 4H_2O$ film deposited on the Nafion membrane during ion- exchange synthesis with the time (a) 10s, (b) 30s, (c) 60s and (d) 180s.
